# Robotic partial nephrectomy for large renal Leiomyoma: first case report

**DOI:** 10.1590/S1677-5538.IBJU.2023.0205

**Published:** 2023-06-20

**Authors:** Antonio Franco, Devin Rogers, Savio D. Pandolfo, Celeste Manfredi, Francesco Ditonno, Ciro Imbimbo, Marco De Sio, Cosimo De Nunzio, Riccardo Autorino

**Affiliations:** 1 Rush University Department of Urology Chicago IL USA Department of Urology, Rush University, Chicago, IL, USA; 2 Sapienza University Sant’Andrea Hospital Department of Urology Rome Italy Department of Urology, Sant’Andrea Hospital, Sapienza University, Rome, Italy; 3 Division of Urology VCU Health Richmond VA USA Division of Urology, VCU Health, Richmond, VA, USA; 4 Federico II University Reproductive Sciences and Odontostomatology Department of Neurosciences Naples Italy Department of Neurosciences, Reproductive Sciences and Odontostomatology, Federico II University, Naples, Italy; 5 “Luigi Vanvitelli” University Urology Unit Naples Italy Urology Unit, “Luigi Vanvitelli” University, Naples, Italy; 6 University of Verona Azienda Ospedaliera Universitaria Integrata Verona Department of Urology Verona Italy Department of Urology, Azienda Ospedaliera Universitaria Integrata Verona, University of Verona, Verona, Italy

## Abstract

**Aim:**

Renal leiomyoma is a rare benign mesenchymal tumor arising from the smooth muscle cells of the kidney. Renal capsule is its most common location (1). Large tumor may require surgical excision which can be challenging in case of proximity to major vessels (2). Indications of robotic partial nephrectomy (RPN) have exponentially expanded over the past few years (3). We aim to report a case of large renal leiomyoma successfully managed with RPN.

**Methods:**

A 59-year-old female patient with BMI 51 presented with chief complaint of abdominal discomfort. The patient underwent a CT scan that revealed a massive circumscribed exophytic complex solid cystic mass of 4.5 × 7.7 × 6.2 cm, arising from the lower pole of right kidney and abutting the inferior vena cava. RENAL score was 11ah (high complexity). Past surgical history included mid-urethral sling, breast reduction, and hysterectomy with salpingectomy. Preoperative creatinine and eGFR were 0.9 (mg/dL) and 77 (mL/min), respectively. A robotic excision of this mass was successfully performed by using Da Vinci Xi platform. Main steps of the procedure are illustrated in the present video.

**Results:**

Dissection and isolation of the tumor were carefully performed after identifying key anatomical structures such as the ureter, the IVC and the renal hilum. Intraoperative ultrasound was used to confirm the margins of the mass. The renal artery was clamped and then the tumor was resected/enucleated. Renal parenchyma was re-approximated with a single layer of interrupted CT-1 Vicryl 0 with sliding clip technique. Warm ischemia time was 19 min. Estimated blood loss (EBL) was 250 ml. Operative time was 165 min. No intraoperative complications occurred. No drain was placed. Patient was discharged on postoperative day 2. Post-operative hypotension was managed with fluid bolus. Postoperative creatinine and eGFR were 1,0 (mg/dL) and 69 (mL/min/1.72m^2^), respectively. Pathology revealed a leiomyoma of genital stromal origin with hyalinization and calcification.

**Conclusions:**

To the best of our knowledge, this is the first description of RPN for the management of a large (about 8 cm) renal leiomyoma. Robotic assisted surgery allows to expand the indications of minimally invasive conservative renal surgery whose feasibility becomes even more clinically significant in case of benign masses which can be managed without sacrificing healthy renal parenchyma.

**Available at:** http://www.intbrazjurol.com.br/video-section/20230205_autorino_et_al

**Int Braz J Urol. 2023; 49 (Video #12): 648-9**
